# Preparation and Mechanical Properties of Graphene Oxide: Cement Nanocomposites

**DOI:** 10.1155/2014/276323

**Published:** 2014-01-16

**Authors:** Fakhim Babak, Hassani Abolfazl, Rashidi Alimorad, Ghodousi Parviz

**Affiliations:** ^1^Civil Engineering and Environment Faculty, Tarbiat Modares University, Tehran 111-14115, Iran; ^2^Nanotechnology Research Center, Research Institute of Petroleum Industry, Tehran, Iran; ^3^Civil Engineering Department, Iran University of Science and Technology, Tehran, Iran

## Abstract

We investigate the performance of graphene oxide (GO) in improving mechanical properties of cement composites. A polycarboxylate superplasticizer was used to improve the dispersion of GO flakes in the cement. The mechanical strength of graphene-cement nanocomposites containing 0.1–2 wt% GO and 0.5 wt% superplasticizer was measured and compared with that of cement prepared without GO. We found that the tensile strength of the cement mortar increased with GO content, reaching 1.5%, a 48% increase in tensile strength. Ultra high-resolution field emission scanning electron microscopy (FE-SEM) used to observe the fracture surface of samples containing 1.5 wt% GO indicated that the nano-GO flakes were well dispersed in the matrix, and no aggregates were observed. FE-SEM observation also revealed good bonding between the GO surfaces and the surrounding cement matrix. In addition, XRD diffraction data showed growth of the calcium silicate hydrates (C-S-H) gels in GO cement mortar compared with the normal cement mortar.

## 1. Introduction

Cementitious materials (especially concrete) are the most common construction materials used worldwide. However, cementitious materials are generally brittle and have very low tensile strength and strain capacity. Macroscopic steel reinforcement bars are commonly used to improve the strength and ductility of this type of material, but in recent decades extensive research on the effects of micro- and macrofibers in controlling the growth of cracks in cementitious materials has been carried out [1, 2]. The concept behind such a transition to fiber-reinforced cement (FRC) is that the resulting tensile strength is developed from many individual fibers rather than a few pieces of steel [1]. Thus, use of discrete fibers results in more uniform distribution of stress within cementitious materials. While microfibers may delay the nucleation and growth of cracks on the microscale, nanoreinforcement would delay the nucleation and growth of cracks on the nanoscale. If nanoscale cracks can be successfully controlled, their propagation to the microlevel would be prevented. Recently, carbon nanostructures such as carbon nanotubes (CNTs, both single and multiwalled), carbon nanofibers (CNFs), and graphene have attracted attention from many concrete researchers because of their exceptional mechanical, chemical, thermal, and electrical properties, and good performance as polymeric reinforcement materials [3, 4]. Graphene is a single layer sp^2^-bonded carbon sheet, which forms a honeycomb crystal lattice. Exfoliated graphene nanoplatelets (xGnP) have the same chemical structure as carbon nanotubes (CNT), and their edges are easily chemically modified for enhanced dispersion in polymeric composites [5]. Such nanoplatelets are typically less than 5 nm thick and can be synthesized with lateral dimensions ranging from <1 to 100 microns. Use of exfoliated graphite flakes could open up many new applications where electromagnetic shielding, electrical conductivity, high thermal conductivity, gas barrier resistance, high fracture toughness, and low flammability are required [5].

Many studies have been carried on the effect of carbon nanotube addition on the cement hydration process and the resulting mechanical properties of the matrix. For example, Makar and Chan reported that CNTs accelerated the hydration process by acting as a matrix for the development of C-S-H and Ca(OH)_2_ [7]. Li et al. found that addition of 0.5% multiwalled CNTs (MWNTs) increased both the 28-day cement mortar compressive and flexural strength of Portland cement composite [8]. Cwirzen et al. obtained a 10% increase of flexural strength for MWNT reinforced composites in comparison with that of plain cement mortar [9, 10]. Konsta-Gdoutos et al. concluded that the optimum concentration of MWCNTs is dependent on their aspect ratio. Short MWCNTs can be used at a high concentration of 0.08 wt%, while long MWCNTs should be used in concentrations lower than 0.048 wt% [11]. Carbon nanofibers have also been reported to provide significant improvements in compression, tensile, and flexural strength when added to macrodefect free cement (MDF) [12]. Sáez De Ibarra et al. studied both single- and multiwalled nanotubes dispersed in distilled water and in water containing gum Arabic to increase the Young's modulus and hardness. They found that single-walled nanotubes were less effective than the multiwalled nanotubes because the single-walled nanotubes were straighter, more defect free, and more difficult to disperse. When gum Arabic was used, the Young's modulus increased for both the multiwalled and the single-walled nanotubes. However, the hardness decreased with respect to that of the plain cement sample [13].

Li et al. experimented with the surface functionalization of multiwalled carbon nanotubes using a 3 : 1 mixture of sulfuric (H_2_SO_4_) and nitric (HNO_3_) acid. They found that the flexural and compressive strengths of 28-day cured cement with carbon nanotubes increased by 25.1 and 18.9%, respectively [14]. In another study Li et al. showed that the compressive and flexural strengths of the treated (functionalized) nanotubes were 2.7 and 0.4 MPa higher than those of untreated nanotubes [15]. Nasibulin et al. have recently developed a method to grow CNTs directly on the surface of cement particles [16]. The use of this cement resulted in a more than 100% increase in the compressive strength of hardened paste. Shah et al. successfully dispersed 0.02 to 0.33 wt% untreated MWCNTs in water containing surfactants by applying optimum ultrasonication and before mixing with cement in water/cement ratios of 0.3 and 0.5. They reported improved mechanical (15 to 55% increase in Young's modulus, 8 to 40% increase in flexural strength) and durability (30 to 40% reduction in autogenous shrinkage) properties [17]. Chaipanich et al. combined 0.5 and 1 wt% CNTs with fly ash cement, observing slight improvements in the resulting compressive strength (around 10%) when compared to cement containing only fly ash [18]. Makar found that at the microscopic level, SWCNT/OPC composites showed evidence of classical reinforcing behavior in the form of crack bridging, fiber pullout, and crack deflection [19]. Sanchez and coworkers studied the effect of CNFs on the mechanical properties of hybrid CNF/silica fume (SF) cement composites. In that study, the addition of CNFs and SF did yield improvement in the compressive or flexural strengths of the cement composite due to CNF and SF agglomeration and bundling [20, 21]. For carbon nanostructures to be fully utilized within a material, they must first be properly dispersed.

The efforts made to date in using carbon nanostructures in cement matrix have been mostly about integrating CNTs/CNFs into cementitious materials. Therefore, in this paper, the effect of another emerging carbon nanostructure, graphene, on the mechanical properties of cement mortar is investigated.

## 2. Experimental Procedures

### 2.1. Materials and Synthesis

Type I Portland cement (Tehran Cement, Iran) mortar was used in this study. Chemical and physical specifications for this type of cement and its allowable ranges in accordance with the National Iranian Standard no. 389 are shown in Tables 1 and 2. Sand used in the cement mortar samples was in accordance with the National Iranian Standard no. 3040, and its gradation and other characteristics are shown in Table 3.

In this study, graphene oxide was synthesized via exfoliation of graphite oxide, performed through a colloidal suspension route [22]. In a typical synthesis process, natural graphite powders were oxidized to graphite oxide using a modified Hummers method [23]. About 1 g graphite powder and 0.5 g sodium nitrate were added to 70 mL concentrated H_2_SO_4_ in an ice bath. Then, 3 g KMnO_4_ was gradually added and the mixture was stirred for 2 h before being diluted with deionised (DI) water. 5% H_2_O_2_ was added to the resulting solution until its colour changed to brilliant yellow, indicating fully oxidised graphite. The as-obtained graphite oxide slurry was redispersed in DI water and then exfoliated by ultrasonication using a Brandson Digital Sonifer (S450D, 35% amplitude) to generate graphene oxide nanoflakes. The mixture was then filtered and washed with diluted HCl solution to remove metal ions. Finally, the product was washed with DI water to remove the acid.

**Figure 1:**
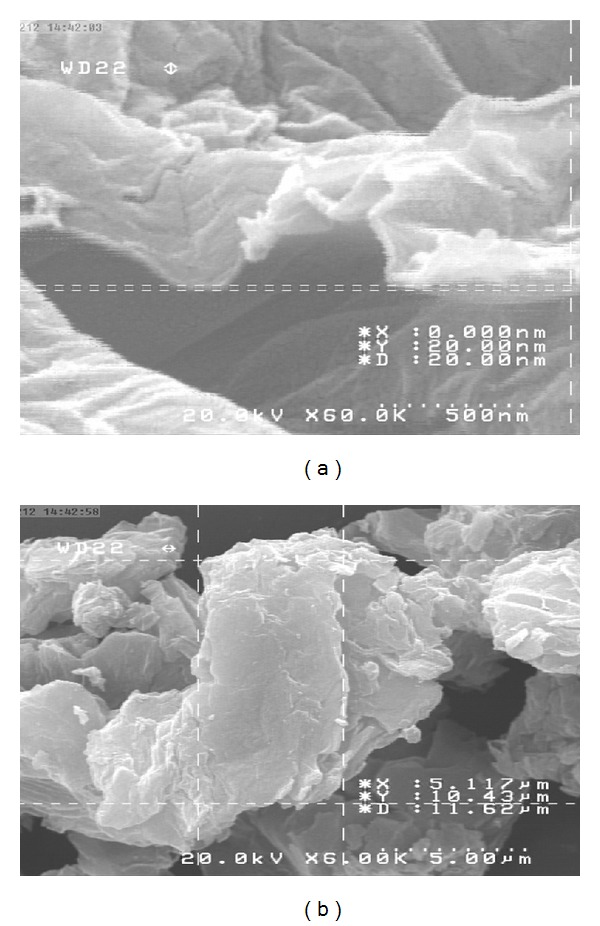
FE-SEM image of aggregated graphene oxide flakes (a). FE-SEM image of graphene oxide flakes with film thickness of about 20 nm (b).

**Figure 2:**
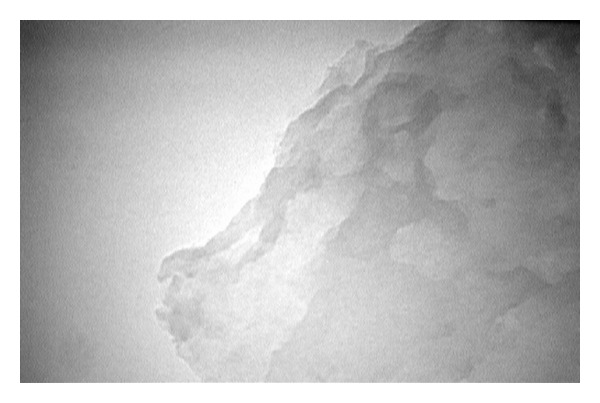
TEM image of graphene oxide (GO) sheets illustrating their flake-like shape.

**Figure 3:**
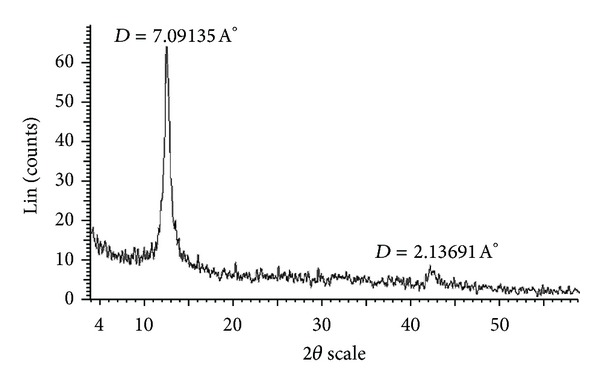
X-ray diffraction patterns of the synthesized nanographene oxide.

**Figure 4:**
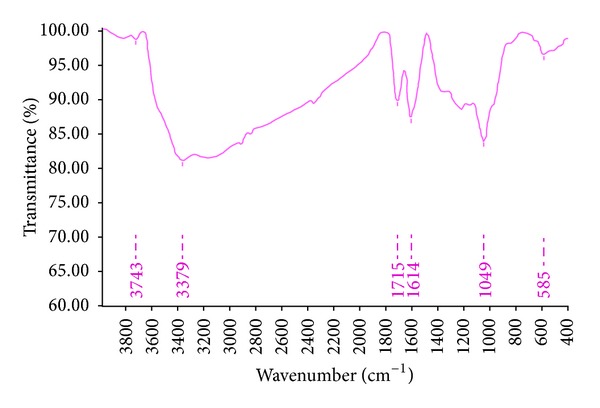
FTIR spectra graph of graphene oxide, showing the presence of different oxygen functionalities.

**Figure 5:**
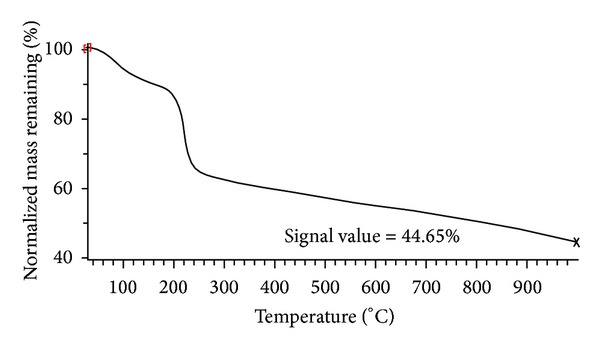
Normalized thermogravimetric analysis (TGA) plots for GO.

Figure 1 shows FE-SEM images of the obtained GO flakes, which had a film thickness of about 20 nm. The GO material consisted of randomly aggregated, thin, crumpled sheets closely associated with each other to form a disordered solid [22]. Transmission electron microscopy (TEM) images illustrating the flake-like shape of the obtained GO are shown in Figure 2.  Figure 3 shows the XRD pattern of the as-synthesized GO. An intense and sharp peak centered at 12.265° corresponded to an interplanar distance of 0.72 nm. The increase in the interplanar distance of GO compared to that of graphite (which has an interplanar distance, d002, of 0.334 nm) is due to the existence of oxygen functional groups and some other structural defects [24].  Figure 4 shows FTIR spectra of the obtained graphene oxide. The presence of different oxygen functionalities in the graphene oxide was confirmed at 3379 cm^−1^ (O–H stretching vibrations), 1715 cm^−1^ (C=O stretching vibrations), 1614 cm^−1^ (skeletal vibrations from unoxidized graphitic domains), 1220 cm^−1^ (C–OH stretching vibrations), and 1049 cm^−1^ (C–O stretching vibrations) [25]. Thermogravimetric analysis (Figure 5) of the graphene oxide (GO) indicated that it was thermally unstable and began to lose mass upon heating even below 100°C. Major mass loss occurred at about 200°C, presumably due to pyrolysis of labile oxygen-containing functional groups to yield CO, CO_2_, and steam [26, 27].

### 2.2. Mortar Mixing

**Figure 6:**
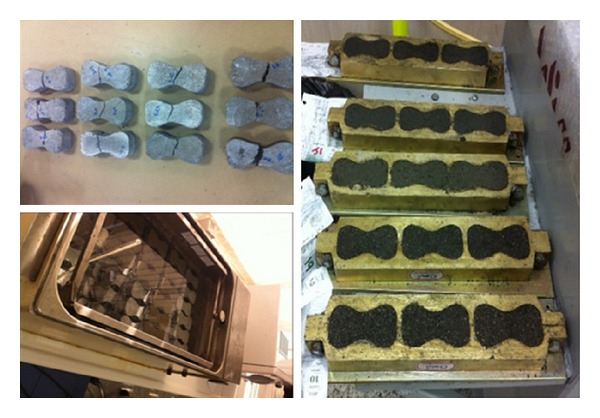
Samples and sample molds used for tensile strength test ASTM C307.

To produce cement mortar containing different percentages of GO and a control sample containing no GO, one part cement and three parts Ottawa standard sand (by weight) were mixed to prepare the mortar samples. Three specimens were fabricated from the same batch for use in tensile strength tests.  Table 4 shows the mix proportions for the samples, which had different GO contents of 0.1, 0.3, 0.5, 1, 1.5, and 2 wt%. The specimens were molded in briquette molds with width and depth of 25 ± 0.5 mm at the waist line, as shown in Figure 6. The specimen materials were left in the mold at the relative humidity of 50% for 24 hours. Before testing, the specimens were cured for a period of 27 days in a water bath at 23 ± 2°C (Figure 5).

### 2.3. Dispersion of Carbon Nanostructures within Cement Matrix

As mentioned earlier, for carbon nanostructures to be fully utilized within a material, they must first be properly dispersed. In the present case, dispersion is the process of separating the bundles of GO into individual flakes within a matrix. To disperse the carbon nanostructures within the cement matrix, the GO was added gradually to water containing polycarboxylate ether (PCE) superplasticizer and the mixture was sonicated for 5 min after each addition to give a total sonication time of 40 min. The sonication conditions were as follows: the amplitude was set to 50%, frequency 20 Hz, power 500 W, titanium alloy probe width 13 mm, and a constant applied energy of 1900 J/min.

After sonication, cement was added to the dispersed GO at a water/cement ratio of 0.4 and mixed for 30 s using a rotary mixer equipped with a flat beater. Sand was then added with mixing, which was then maintained for a further 3.5 min. This process followed the ASTM C 109 procedure (ASTM C109/C109 M, 2008).

### 2.4. Tensile Strength Test

Standard test ASTM C307-03 was used to investigate the tensile strength of cement mortar containing different percentages of GO in comparison with control samples containing no GO. After curing, the depth and width at the waist of each test specimen were measured to the nearest ±0.5 mm. Then, the specimens were loaded in a tensile testing machine at a crosshead speed of 5 to 6.4 mm/min (speed when the machine ran without a load).

### 2.5. SEM Characterization

SEM (FE-SEM; Hitachi S416) was used to observe the dispersion and bonding properties between the nano-GO flakes on fracture surfaces of the cement mortar. After the 28-day cured samples had been tested, the fracture surface was cut into an approximately 10 × 5 × 4 mm sample, and coated with a 3 nm-thick platinum/palladium layer to enhance its conductivity.

### 2.6. Composition

The composition of the cement matrix was determined in two ways. Energy dispersive analysis by X-ray (EDAX; TESCAN VEGA XMU) was performed to determine the constituent elements of the material and XRD was performed to determine the crystalline phases of the material. Quantitative analysis of the crystalline and amorphous phases of hydrated cementitious nanocomposites can be carried out using the internal standard XRD technique. In this study, the crystalline phase was determined using a Bruker AXS D8 Advance X-ray diffractometer (XRD) employing a scanning rate of 0.02°/s in a 2*θ* range of 4–70° with CuK*α* radiation (*λ* = 1.540 Å) and an accelerating voltage of 40 kV and current of 30 mA. Phase identification was accomplished with search-match software using the International Centre for Diffraction Data (ICDD) database (International Center for Diffraction Data, Newtown Square, PA, USA).

## 3. Results and Discussion

**Figure 7:**
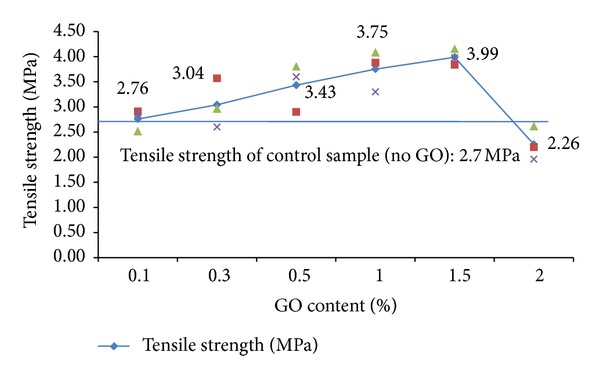
Tensile strength results of cement mortar specimens with graphene oxide content.

The results of the tensile strength tests are shown in Figure 7. The tensile strength of the specimens was observed to increase with nano-GO percentage until it reached 1.5%, after which a decrease in tensile strength was observed for 2 wt% GO content. As shown in this figure, specimens containing 1.5 wt% GO flakes exhibited about a 48% increase in tensile strength compared with that of the control mortar samples.

In the literature, it has been concluded that small amounts of carbon nanostructures (such as MWCNTs) of about 0.03–0.1 wt% give the best gains in the mechanical properties of cementitious nanocomposites [17]. However, in this paper the best result was obtained at 1.5 wt% GO content. This could be due to better dispersion of GO compared with that achievable with other nanocarbon structures such as MWCNTs. Dispersion of this kind of nanoflake within a material matrix is very difficult because CNTs and CNFs attract each other because of van der Waals forces, which result in the formation of agglomerations (bundles) in the form of entangled ropes and clumps. Another factor which can cause problems in dispersing such nanofilaments (CNTs, CNFs) within a cement matrix is their too large aspect ratio. Konsta-Gdoutos et al. concluded that the optimum concentration of MWCNTs in the matrix generally depends on their aspect ratio. Short MWCNTs can be used in a higher concentration, while long MWCNTs should be used in lower concentrations [11]. Therefore, the plate form (small aspect ratio) and oxygen functionalities (such as polycarboxylate) of the present GO makes it easier to disperse, and subsequently its optimum percentage was higher than that reported for other carbon nanostructures such as CNTs and CNFs.

To successfully apply carbon nanostructures as cement matrix reinforcement, two key requirements must be met: good dispersion and optimal bond strength [3].

Chemical alteration of the flake surface through creation of noncovalent bonds with surfactants is used to disperse nanoflakes and to maintain long-term suspension in a variety of liquid solutions. Surfactants are wetting agents that are used to lower the surface tension of water, and can therefore allow easier dispersion of carbon nanostructures [28, 29]. Ultrasonic mixing, which uses a high frequency driver to transmit acoustic energy through a liquid medium, is then used to separate nanofilament bundles in water containing surfactants or other solvents.

**Figure 8:**
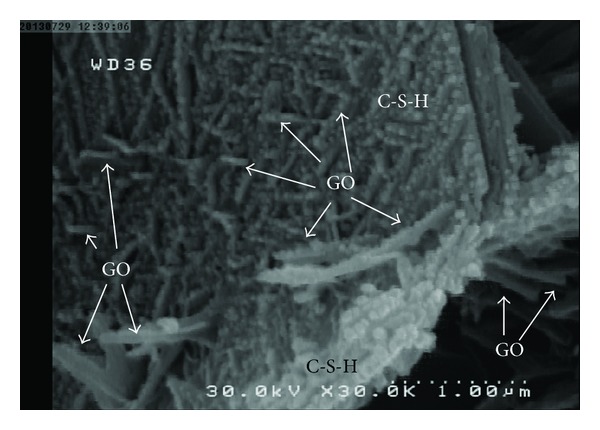
FE-SEM images of cement mortar containing 1.5 wt% GO at a scale of 1.0 *μ*m after 28 days curing.

Figure 8 shows FE-SEM images of a cement mortar containing 1.5 wt% GO at a scale of 1.0 *μ*m. The nano-GO flakes were found to be well dispersed and there were no GO agglomerates visible in the matrix. Thus, the first requirement was achieved.

**Figure 9:**
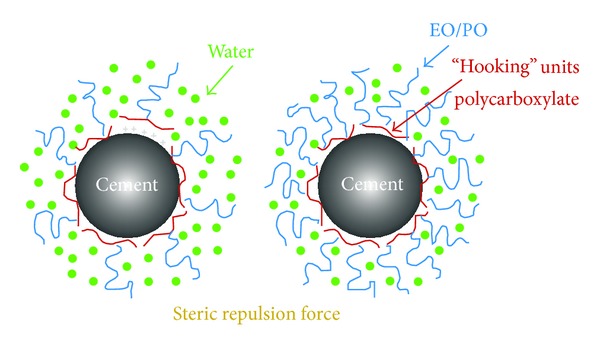
The Mechanism of steric repulsion of comb polymer in dispersion [6].

Graphitic nature of GO makes it very difficult to achieve proper adhesion with a cementitious. Bonding between the cement matrix and flakes is very weak, which causes them to slide out of the cement matrix under a load much lower than the strength of the individual carbon nanostructures. This sliding becomes more pronounced within the bundles if the nanoflakes lack proper dispersion. To make use of as much of the mechanical properties of the carbon nanostructures as possible, interfacial bonding and frictional properties need to be optimized. Polycarboxylate superplasticizer (0.5 wt% of cement) was used to improve the adhesion properties of GO and its dispersion in the cement matrix. Superplasticizer additives based on polycarboxylate (COOH) are one of the best surfactants for dispersing carbon nanostructures within a cement matrix [6, 28, 30]. On the other hand, as shown by the FTIR spectra, different types of oxygen functionalities existed in the graphene oxide (Figure 4). These oxygen functionalities (such as polycarboxylate) also facilitated dispersion of the GO flakes within the cement matrix. The electrostatic repulsion force for polycarboxylate based superplasticizers is only half the value measured for conventional water-reducing admixtures, the dispersion mechanism of which can be explained in terms of electrostatic repulsion between cement particles. In contrast, for polycarboxylate based superplasticizers dispersion is mainly caused by a very strong steric hindrance effect that “pushes” the cement particles apart. These steric repulsion forces are caused by polyoxyalkylene pendant groups attached to the backbone of PC based superplasticizers, as illustrated in Figure 9. Such water reducing admixtures cause functional groups (COOH) to attach to the surface of GO (increased functionalization), which reduces van der Waals forces between the GO flakes and increases the energy required to pull them from the cement matrix, thereby improving bonding characteristics between the nano-GO and the cement matrix.

**Figure 10:**
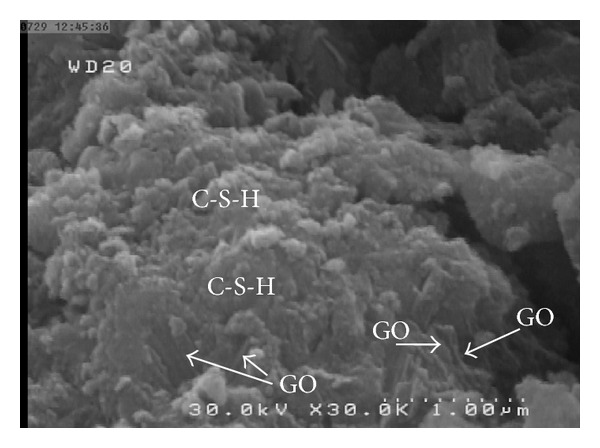
FE-SEM images of cement mortar containing 1.5 wt% GO at scales of 1.0 *μ*m after 28 days curing, showing calcium silicate hydrates (C-S-H) gel in the form of a dense sponge matrix that gradually spread, merge, and adhere to GO, strengthening the cement and reducing its permeability.

Figure 10 shows FE-SEM images of a cement mortar containing 1.5 wt% GO at a scale of 1 and 5 *μ*m. As shown in this image, calcium silicate hydrates (C-S-H) gels, which are the most desirable product of cement hydration and greater contributor to cement matrix strength and low permeability, existed in the form of a dense sponge matrix that gradually spread, merged, and adhered to the GO, strengthening the cement and reducing its permeability. With sufficient hydration, C-S-H gel forms a solid mass [31].

**Figure 11:**
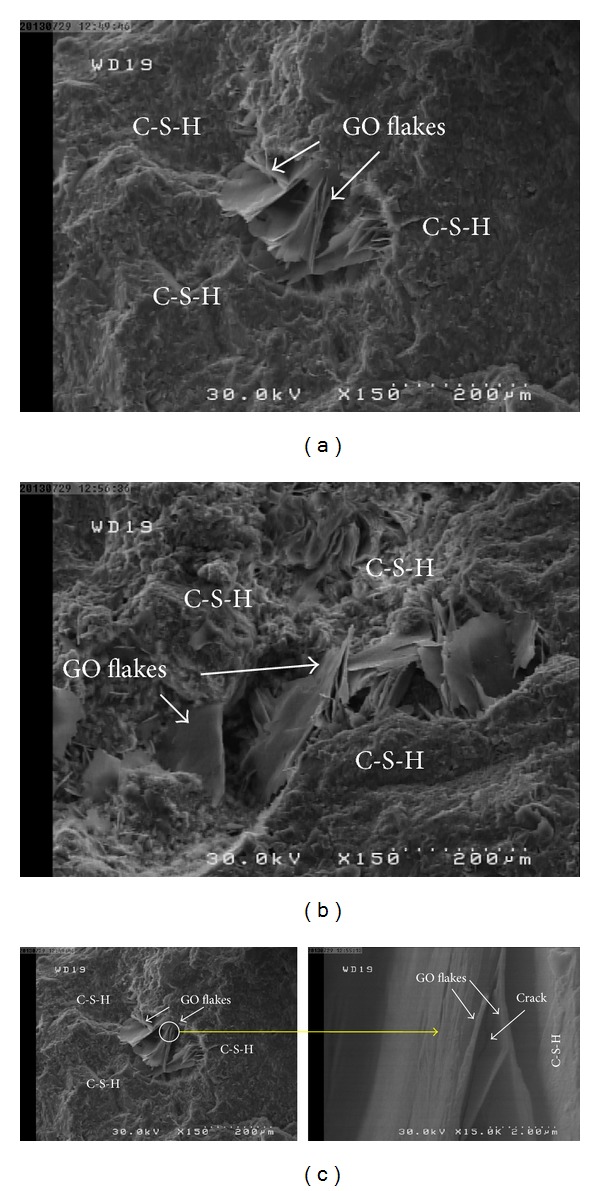
FE-SEM images of cement mortar containing 1.5 wt% GO at scales of 200 *μ*m after 28 days of curing, showing the nucleation of C-S-H by the GO flakes and its formation along them ((a) and (b)). FE-SEM images of cement mortar containing 1.5 wt% GO at scales of 200 and 2.0 *μ*m after 28 days curing, showing microcracks on a GO flake under tensile stresses (c).

Another mechanism which causes stronger bonding between GO flakes and the cement matrix is a nucleating effect of the graphene oxide flakes. Thus, the main reason for the high bonding strength appeared to be due to the nucleation of C-S-H by the GO flakes and its formation along them (Figure 11). The hydrated cement products deposited on the GO flakes due to their higher surface energy and the presence of hydrophilic groups on the GO surfaces acted as a nucleation site. Nucleation of hydration products on nanoparticles further promotes and accelerates cement hydration [32, 33]. The addition of colloidal silica resulted in acceleration of silicate phases (alite (C_3_S) and belite (C_2_S)) dissolution and rapid formation of C-S-H phase in cement matrix [32]. Thus, GO flakes acted as nucleating agents for C-S-H, which preferentially formed on the surface of the GO flakes instead of on the surface of the adjacent unhydrated cement grains.

**Figure 12:**
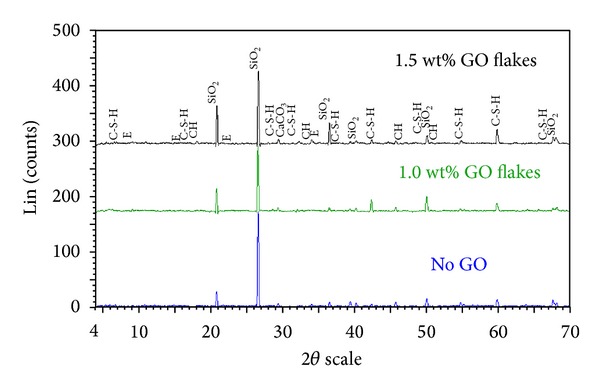
X-ray diffractogram of GO-cement nanocomposites containing 1 and 1.5 wt% GO flakes and control sample (without any GO flakes) after 28 days of curing.

**Figure 13:**
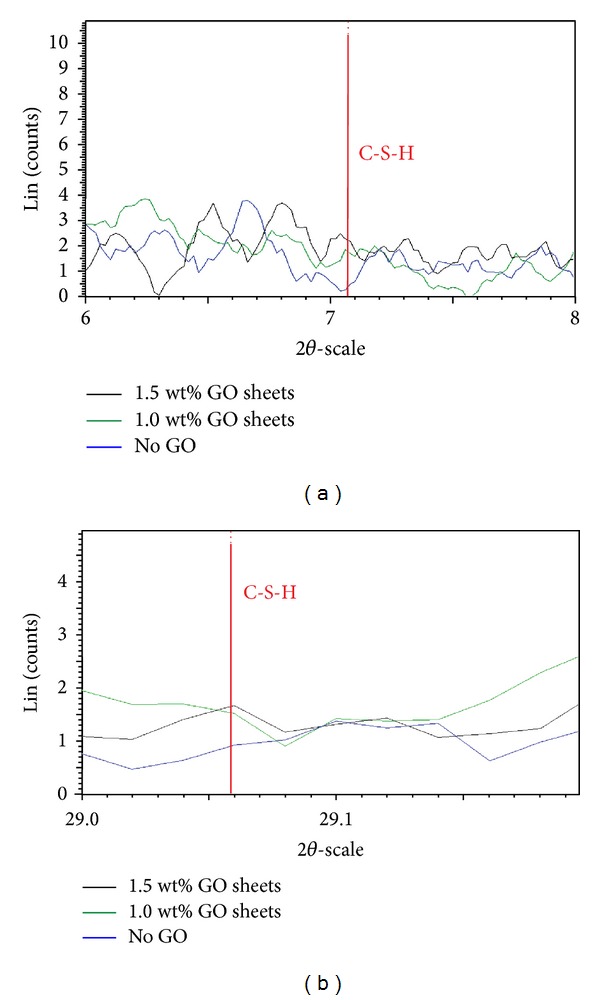
Comparison of X-ray diffraction data obtained from GO-cement nanocomposites containing 1 and 1.5 wt% GO flakes and control sample (without any GO flakes) after 28 days (enlarged view).

**Figure 14:**
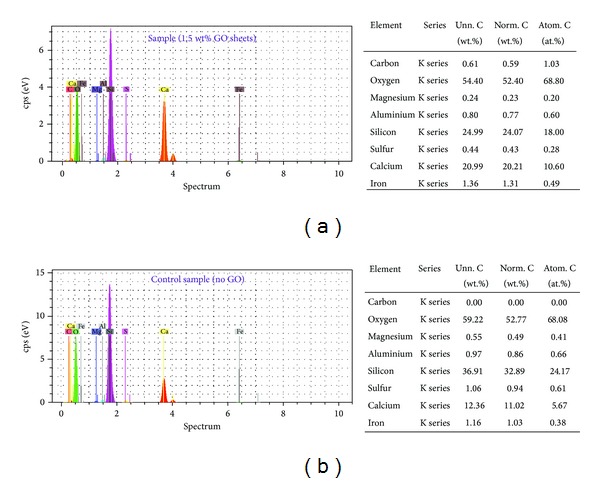
Composition of the GO-cement nanocomposites containing 1.5 wt% GO flakes (a) and control sample (b) (without any GO flakes) after 28 days, measured by energy dispersive analysis by X-ray (EDAX).

Figures 12 and 13 show a comparison of XRD data obtained for GO-cement nanocomposites containing 1 and 1.5 wt% GO flakes and the control sample (without any GO flakes) after 28 days. While Figure 12 shows no major changes in the crystallinity, Figure 13 (a closer look) shows growth of the calcium silicate hydrates (C-S-H) gels compared with the normal cement mortar. It can be because of the nucleation of C-S-H by the GO flakes which was shown in Figure 11. It is worthy to mention that although the C-S-H which is formed in cement hydration is amorphous, the powder diffraction pattern of 11 Å tobermorite (ICDD no. 34-0002) can resemble the poorly-crystalline product (C-S-H) formed upon hydration of Portland cement [34, 35]. Because of the small percentages of GO content used and other instrumental limitations, no peak corresponding to graphitized carbon appeared in the XRD patterns of the GO-cement. Therefore, EDAX was used to confirm the presence of graphitized carbon in the GO reinforced cement composites (Figure 14).

The FE-SEM images of Figure 12 show a microcrack in a GO flake. Since the crack was aligned perpendicular to loading, it can be concluded that the Go flake had been under the tensile stresses. This implied good bonding between the GO surfaces and the surrounding cement matrix. The breakage seen in the image indicated that very high stresses had been applied to the GO flakes. Because the theoretical tensile strength of GO flakes is very high (about 130 GPa), more GO flakes are needed in order to carry stresses [5]. The fact that breakage, not pullout, is seen in the images implies that good dispersion is the real key to improving the mechanical properties of cement nanocomposites rather than bonding between the GO and the matrix. This may be the main reason that even the tensile strength of samples which have the least GO content (0.1 wt %) is higher than that of the control mortar samples which have no GO at all.

**Figure 15:**
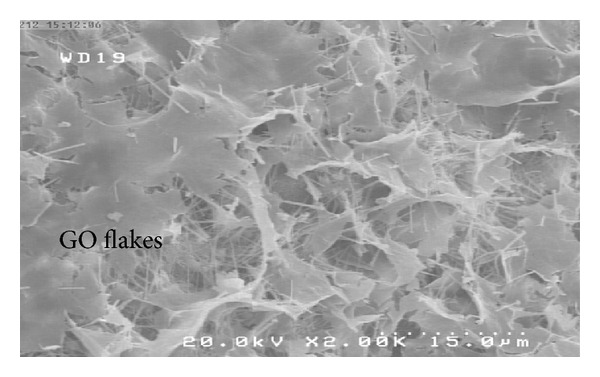
FE-SEM images of cement mortar containing 2.0 wt% GO at a scale of 15 *μ*m.

On the other hand, increasing GO content while the water/cement ratio of matrix is held constant causes difficulty in providing suitable workability and consequently dispersing GO within the matrix due to the presence of hydrophilic groups on the GO surfaces. In this circumstance, as shown in Figure 15, nano-GO flakes absorb a nonnegligible amount of water, hampering the hydration of the cement mortar and also causing them to agglomerate in the form of clumps which are very difficult to disentangle. These agglomerates form large voids within the cement matrix and stresses cannot be transferred across the bundles. In addition, if the GO bundles remain intact, they no longer remain within the nanoscale range. Instead of filling the nanosized void spaces within the cement grains, they gather between cement hydration products and create zones of weakness throughout the cement matrix. This could be the main reason why the tensile strengths of specimens containing 2 wt% GO were much lower than those of the control samples.

To confirm this hypothesis, we prepared new cement mixtures, with and without GO, and increased the water/cement ratio to 0.50, following the same procedure previously described. After 28 days of curing, the tensile strength of the control and 2 wt% GO containing samples was equal to 2.4 and 2.99 MPa, respectively (average of three samples each). Obviously, due to a higher water/cement ratio, the tensile strength of control cement mortar was lower for these samples with respect to the previous ones, but in this higher w/c ratio, the GO loading used (2 wt%) led to the 24.7% increase in mechanical strength (Table 5).

## 4. Conclusions

In this study, GO was synthesized via exfoliation of graphite oxide prepared by a colloidal suspension route and was used to prepare GO-cement nanocomposites (GCNC) using an ultrasonic method. A polycarboxylate super plasticizer (0.5 wt% of cement) was used to improve the adhesion properties of the GO and uniformly disperse it in the cement matrix. Use of an optimal percentage (1.5 wt%) of GO nanoplatelets caused a 48% increase in the tensile strength of the cement mortar specimens. Moreover, using FE-SEM observation of the fracture surface of the samples containing 1.5 wt% GO revealed that the GO nanoplatelets were well dispersed and no GO agglomerates were seen in the matrix. In addition, XRD data shows growth of the calcium silicate hydrates (C-S-H) gels in GO cement mortar compared with the normal cement mortar. It can be because of the nucleation of C-S-H by the GO flakes which was shown in FE-SEM images. The hydrated cement products deposited on the GO flakes due to their higher surface energy and the presence of hydrophilic groups on the GO surfaces acted as a nucleation site. The results indicated that the main reason for the observed high bond strength was the nucleation of C-S-H by the GO flakes and its formation along them. FE-SEM observation also revealed microcracks in the GO flakes, implying that the GO flakes stretched across microcracks in the mortar. The breakage observed indicated that very high stresses were applied to the GO flakes. Because the theoretical tensile strength of GO flake is very high, more GO flakes are needed to carry stresses. The tensile strength of specimens containing 2 wt% GO flakes was much less than that of the control samples. This behavior was justified by taking into account that GO was hydrophilic enough to absorb most of the water contained in the cement mortar, hampering the proper hydration of the cement mortar and making dispersion of the GO within the matrix difficult. This hypothesis was confirmed by the 24.7% increase obtained in the tensile strength of specimens containing 2 wt% GO at a water/cement ratio of 0.5 compared with that of the sample containing 2.0 wt% GO at a water/cement ratio of 0.4.

## Figures and Tables

**Table 1:** Chemical contents of type I cement, according to national standard no. 389 (Tehran Cement).

Constituent compounds	CaO, %	SiO_2_, %	Al_2_O_3_, %	Fe_2_O_3_, %	MgO, %	SO_3_, %	L.O.I, %	I.R, %
Measured value	62.35	21.45	4.61	3.3	3.26	2.05	2.00	0.57

**Table 2:** Properties of type I cement type I, according to national standard no. 389 (Tehran Cement).

	Chemical properties			Physical properties						
	MgO	L.O.I	I.R	Blaine specific surface cm^2^/g	Autoclave expansion, %	Setting time		Compressive strength kg/cm^2^		
	%	%	%			Initial minutes	Final hours	2 days	At least 28 days	At last 28 days
Value	<5	<3	<0.75	>2800	<0.8	>45	<6	>100	>425	<625

**Table 3:** Gradation of standard sand, according to national standard no. 3040.

Square mesh size (mm)	Remaining on the sieve (%)
2.00	0
1.60	7 ± 5
1.00	33 ± 5
0.5	67 ± 5
0.16	87 ± 5
0.08	99 ± 1

**Table 4:** Mixture proportions for the samples.

Graphene, %	Water : cement ratio	Cement weight, g	Water weight, g	GO weight, g	Sand weight, g	Additive weight, g	Total weight, g
0	0.4	147.56	59.02	0.00	442.68	0.74	650
0.1	0.4	147.51	59.06	0.15	442.54	0.74	650
0.3	0.4	147.42	59.14	0.44	442.26	0.74	650
0.5	0.4	147.33	59.22	0.74	441.98	0.74	650
1	0.4	147.09	59.43	1.47	441.28	0.74	650
1.5	0.4	146.86	59.62	2.20	440.58	0.73	650
2	0.4	146.63	59.82	2.93	439.88	0.73	650

**Table 5:** The comparison between the tensile strength results of samples at the different w/c ratio.

GO content, %	Average Tensile strength (MPa)		Percentage change
	W/C = 0.4	W/C = 0.5	
0.0	2.7	2.40	−11.11
2.0	2.26	2.99	24.70
